# Draft Genome Sequence of *Myxococcus xanthus* Wild-Type Strain DZ2, a Model Organism for Predation and Development

**DOI:** 10.1128/genomeA.00217-13

**Published:** 2013-05-09

**Authors:** Susanne Müller, Jonathan W. Willett, Sarah M. Bahr, Cynthia L. Darnell, Katherine R. Hummels, Carolyn K. Dong, Hera C. Vlamakis, John R. Kirby

**Affiliations:** Department of Microbiology, University of Iowa, Iowa City, Iowa, USAa;; University of Chicago, Department of Biochemistry and Molecular Biology, Chicago, Illinois, USAb;; U.S. Department of State, Bureau of Economic and Business Affairs, Office of Agriculture, Biotechnology, and Textile Trade Affairs, Washington, DC, USAc;; Harvard Medical School, Department of Microbiology and Immunobiology, Boston, Massachusetts, USAd

## Abstract

*Myxococcus xanthus* is a member of the *Myxococcales* order within the *Deltaproteobacteria* subdivision. The myxobacteria reside in soil, have relatively large genomes, and display complex life cycles. Here, we report the whole-genome shotgun sequence of strain DZ2, which includes unique genes not found previously in strain DK1622.

## GENOME ANNOUNCEMENT

*Myxococcus xanthus* is a member of the *Myxococcales* order (fruiting and gliding bacteria) within the *Deltaproteobacteria* subdivision. The myxobacteria typically reside in soil, are characterized by relatively large genomes (up to ~13 Mb), and display complex life cycles. In response to a variety of environmental cues, *M. xanthus* organizes into multicellular fruiting bodies harboring stress-resistant spores. Fruiting body formation is regulated by several mechanisms involving complex signal transduction cascades, production of cytosolic and extracellular signals, and gliding motility (1–34). Previous work determined the sequence of the *M. xanthus* laboratory strain DK1622 (accession number NC_008095.1) (34). While both DK1622 and the closely related strain DZ2 originate from the Roger Y. Stanier collection originally housed at the University of California, Berkeley, strain DK1622 was genetically modified from a parent (35). Moreover, phenotypic differences between DZ2 and DK1622 regarding rates of autolysis, rippling behavior, aggregation, and fruiting body formation have been reported (9, 36, 37).

*M. xanthus* strain DZ2 was sequenced at the University of Iowa DNA Core Facility using 454 GS-FLX titanium technology. Genomic DNA was prepared by resuspending cell pellets in SET buffer (75 mM NaCl, 10 mM Tris [pH 7.5], 25 mM EDTA, 1% SDS, and 1 mg/ml proteinase K) and then incubating the suspension for 2 h at 55°C. DNA samples were subsequently extracted with chloroform (3×), precipitated with isopropanol, and resuspended in 10 mM Tris (pH 8.0). The sample was processed for 454 sequencing according to established protocols. The resulting sequence represents approximately 20-fold coverage. The genome was assembled *de novo* using Newbler software version 2.7. The sequence comprises 292,633 reads totaling 186 Mb and was assembled into 87 contigs. Using the RAST genome annotation server, we were able to predict a total of 7,709 coding sequences (CDS) within the *M. xanthus* DZ2 genome (38).

The genome of DZ2 is 9.287 Mb, approximately 196 kb larger than the published genome of *M. xanthus* strain DK1622. A similar size differential was described previously and is presumed to be the result of UV mutagenesis on the DK1622 progenitor strain DK101 (39, 40). The vast majority of the DZ2 genome is identical to the DK1622 genome, with the notable exception of DZ2-specific genes. Many DZ2-specific genes encode hypothetical proteins with high homology to sequences found within other myxobacteria, including *Myxococcus fulvus, Stigmatella aurantiaca*, and *Sorangium cellulosum*. Importantly, we identified unique genes likely to encode proteins involved in regulation of transcription or translation, signal transduction, fatty acid modification, and protein transport. The presence of DNA sequences unique to DZ2 has been verified by PCR. We are currently investigating whether the reported phenotypic differences between strains DZ2 and DK1622 might be attributable to production of unique proteins.

### Nucleotide sequence accession numbers.

This whole-genome shotgun project has been deposited at DDBJ/EMBL/GenBank under the accession number AKYI00000000, corresponding to BioProject PRJNA168264. The version described in this paper is the first version, AKYI02000000.
